# Prevalence and Associated Factors of Hypertension: A Community-Based Cross-Sectional Study in Municipalities of Kathmandu, Nepal

**DOI:** 10.1155/2016/1656938

**Published:** 2016-05-12

**Authors:** Raja Ram Dhungana, Achyut Raj Pandey, Bihungum Bista, Suira Joshi, Surya Devkota

**Affiliations:** ^1^Nepal Family Development Foundation, Kathmandu, Nepal; ^2^Nepal Health Research Council, Kathmandu, Nepal; ^3^Ministry of Health and Population, Kathmandu, Nepal; ^4^Manmohan Cardiothoracic, Vascular and Transplant Centre, Institute of Medicine, Tribhuvan University, Kathmandu, Nepal

## Abstract

*Objective*. This study aimed to assess the prevalence and associated factors of hypertension in newly declared municipalities of Kathmandu, Nepal.* Design, Settings, and Participants*. This was a community-based cross-sectional study conducted in the municipalities of Kathmandu District, Nepal, between January and July 2015. Study participants were aged 18 to 70 years, residing permanently in the study sites. Municipalities, Wards, households, and respondents were selected randomly.* Results*. Of the 587 participants, 58.8% were females, mean (SD) age was 42.3 (13.5) years, 29.3% had no formal education, 35.1% were Brahmins, and 41.2% were homemakers. Prevalence of hypertension was 32.5% (95% CI: 28.7–36.3). Age, gender, education, ethnicity, occupation, smoking, alcohol consumption, physical activity, diabetes, menopausal history, and family history of cardiovascular disease (CVD) and hypertension were significantly associated with hypertension. In multivariable analysis, smoking, alcohol consumption, physical activity, body mass index, and diabetes were identified as significant explanatory variables for hypertension.* Conclusion*. This study demonstrated that the people living in newly established municipalities of Kathmandu, Nepal, have a high burden of hypertension as well as its associated factors. Therefore, community-based preventive approaches like lifestyle modification and early detection and treatment of hypertension might bring a substantial change in tackling the burden effectively.

## 1. Background

Worldwide levels of hypertension represent the global public health crisis. Hypertension affects around 22% of people aged 18 years and over and is responsible for an estimated 9.4 million deaths per year globally [1, 2]. Raised blood pressure mostly remains asymptomatic while increasing the risk of heart disease, stroke, and renal failure [3]. It contributes to at least 45% of deaths due to heart disease and 51% of deaths as a result of stroke [1]. Additionally, around 10% of healthcare expenditure is directly related to hypertension and its complications [3].

The increasing prevalence of hypertension in developing countries is a major concern. According to recent estimates from the World Health Organization, two-thirds of hypertensive people live in developing countries [4]. Africa has the highest prevalence of hypertension (29.6%) followed by the Eastern Mediterranean (26.9%), South East Asia (24.7%), Europe (23.3%), the Western Pacific (18.7%), and America (18.2%) [2]. Among South Asian countries, Nepal reported the second highest proportion of hypertensive people (27.3%) after Afghanistan (29%) [2].

There are a range of factors that increase the risk of developing hypertension [5]. Tobacco use increases the incidence of cardiovascular diseases including hypertension [6]. Alcohol consumption is related to 5% to 30% of hypertension cases in the general population [7, 8]. Similarly, about 30% of cases are attributed to increased salt consumption and 20% to physical inactivity [9]. Hypertension has also well-established relationship with obesity and diabetes [10, 11]. Recent reports have provided evidence that increasing rates of noncommunicable diseases, including hypertension, are associated with other determinants like increases in rapid unplanned urbanization, globalization, and sociodemographic and nutritional transition [12, 13].

The surroundings of Kathmandu metropolitan city have many of the characteristics that can lead to an increase in noncommunicable diseases or conditions like hypertension. These factors include high internal migration from other regions of Nepal, increasing sedentary lifestyles, inadequate development and planning, high levels of air pollution, and changes in the dietary habits of residents [14]. However, there is little evidence on the prevalence and risk factors of hypertension in the periurban areas of Kathmandu District. Therefore, this study was conducted to estimate the prevalence of hypertension and to quantify health and social determinants related to hypertension in periurban areas of Kathmandu.

## 2. Methods

### 2.1. Study Design, Settings, and Participants

This was a community-based, cross-sectional study conducted in the newly established (established in December 2014) municipalities of Kathmandu District, Nepal, between January and July 2015. The ten municipalities are located on the outskirts of Kathmandu metropolitan city and are areas with rapid, unplanned urbanization and with high immigration of people from other areas of Nepal [14]. These municipalities contain a total population of more than 300,000 people [15].

Study participants were aged between 18 and 70 years, holding permanent resident status in the study areas at the time of study. Individuals diagnosed with any mental disorder or pregnant women were excluded from the study.

Of the ten municipalities in Kathmandu, two municipalities were randomly selected, one each from the eastern (Kageshwari-Manohara) and western (Nagarjun) clusters. These municipalities consist of around 130,000 people living in 27 Wards. From the total Wards, four Wards, two from each municipality, were randomly selected. The sample was proportionately allocated to each Ward based on the number of households in the Ward. A systematic random sampling method was then applied to choose 600 households from around 5600 households. The study area was mapped and each household was assigned a number, beginning from clockwise direction from first lane of eastern corner of the sites. One household was randomly selected from the first 10 houses using random number table, and then every 10th household from that initial household was approached systematically until the sample size was completed. Among adult members of each household, one individual from that household was enrolled in the study by KISH method [16].

### 2.2. Data Collection

Data were collected by face-to-face interview, anthropometric measurements, and clinical examinations. Fifteen medical professionals were recruited and trained in data collection methods.

#### 2.2.1. Interview

The survey questionnaire (WHO STEPwise questionnaires) covered the demographics and health behaviors of respondents [17]. Demographic information included age, sex, ethnicity, marital status, years at school, and primary occupation. The health behaviors included tobacco use, alcohol consumption, fruit and vegetable consumption, and physical activity. 

*Tobacco Use*. Questions were asked to identify current users, daily users, and past users. 

*Alcohol Consumption*. Questions were asked to determine the current users of alcohol. Detailed information, such as the number of standard drinks consumed in the last 30 days, was obtained from current users. Pictorial cards showing different kinds of glasses and bowls most commonly used in Nepal were used to help the participants recall the amount of alcohol consumed. The amount, as identified by the respondent, was then used to estimate the number of standard drinks of alcohol consumed (one standard drink being defined as 10 grams of ethanol). 

*Diet*. Seventy-two hours dietary recall was used to estimate fruit and vegetable consumption. Determination of the amount of fruit and vegetables consumed was aided by pictorial show cards and standard measuring cups. 

*Physical Activity*. Seven-day history of physical activity was recorded. The physical activities related to work, transport, and recreational activities were categorized into vigorous, moderate, and low levels of activity in accordance with published guidelines [18, 19]. Vigorous physical activity was defined as any activity that had more than six Metabolic Equivalent of Task (MET) values. Moderate physical activity was defined as any activity that had MET values between three and six [18]. Physical activity having less than three METs, like spending time sitting at a desk or travelling in a car, bus, or train, was considered low or sedentary physical activity. 

*Diabetes*. Participants were requested to provide information on status of diabetes or diabetic medication history. 

#### 2.2.2. Anthropometric Measurement

Height and weight were measured using the digital weighing machines and the portable standard stature scales, respectively, from which body mass index (BMI) was calculated. Waist and hip circumferences were also measured by Johnson's nonstretchable measuring tape in order to determine the waist-hip ratio.

#### 2.2.3. Clinical Examination

Trained data collectors measured blood pressure using an aneroid sphygmomanometer. Before taking the measurements, respondents were requested to sit quietly and rest for 15 minutes with legs uncrossed. Three readings of the systolic and diastolic blood pressure were taken with three-minute rest between each reading and the mean of second and third reading was used for analysis.

#### 2.2.4. Operational Definitions

Standard operational definitions were adopted for key variables to maintain consistency and uniformity of the information. 

*Poor*. Poor was defined as those having income less than 40,933 Nepalese Rupees per annum [20]. 

*Current Smoker*. Current smokers were defined as those who reported smoking any tobacco product within last 30 days. Respondents who reported smoking at least 100 cigarettes in their lifetime and who, at the time of the survey, did not smoke were defined as past smoker [21]. 

*Current Alcohol Drinkers*. Those who consumed alcohol within last 30 days were considered current alcohol drinkers [21]. 

*Sufficient Fruit and Vegetables Intake*. Intake of at least 400 gm of fruit and vegetables in a day was regarded as sufficient [22]. 

*Sufficient Physical Activity*. Sufficient physical activity was defined as ≥600 METs of moderate and vigorous activity in a week [18]. 

*Hypertension*. The diagnostic criterion for hypertension was set as a systolic blood pressure ≥ 140 mmHg and/or a diastolic blood pressure ≥ 90 mmHg as recommended by Joint National Committee-VII. Those research participants who were using antihypertensive medicine were also considered as hypertensive [23]. 

*Diabetes*. Self-reported status or the use of any antidiabetic medication was the diagnostic criteria for diabetes. 

### 2.3. Sample Size

Sample size was calculated for a single sample of the estimated population using the specified absolute precision formula (*N* = *z*2*pq*/*d*2) [24]. For estimation of sample size, prevalence of hypertension was taken as 25.7% [25] with 5% of allowable error. The sample size was adjusted with design effect (multiplied by 2) and nonresponse rate (2%). The total calculated sample size was 600.

### 2.4. Data Management and Analysis

Data were compiled, edited, and checked to maintain consistency. Duplications and omissions of data were corrected before coding and entering them in Epidata V.2.1. Data were then exported to SPSS V.16.0 for analysis.

Frequencies and percentages were calculated to identify the distribution of sociodemographic characteristics. Chi-square and independent *t*-tests were conducted for comparing proportions of categorical and mean of continuous variables. Nonnormally distributed data were analyzed by Mann-Whitney *U* test. For multivariable analysis, hypertension was considered as a dichotomous dependent variable. Study variables having significant association with hypertension in bivariate analysis were entered in logistic model using Stepwise Forward Conditional method. The result was validated using Backward Conditional method. Variables having collinearity and confounding effects (waist circumference and hip circumference with BMI; age with diabetes) were excluded from the analysis. All tests were two-tailed and *p* < 0.05 was considered statistically significant.

## 3. Results

### 3.1. Sociodemographic Characteristics

The total number of study participants was 587, excluding 13 who did not respond well. Of these, 58.8% were females. The mean (SD) age of participants was 42.3 (13.5) years. Nearly one-third (29.3%) of participants had no formal education. Of total participants, Brahmins comprised the largest proportion (35.1%), followed by Chetris (29.1%) and Newars (20.3%). Nearly half of the respondents were homemakers (41.2%) and were living below the poverty line (49%).

The difference in male and female count was not statistically significant by age group, ethnicity, and income level. However, education level and occupation significantly varied by gender (Table 1).

### 3.2. Prevalence of Hypertension

The prevalence of hypertension in the study population was 32.5% (95% CI: 28.7–36.3). However, only 15.8% of participants were taking antihypertensive medication. Half of them (53.8% of hypertensive participants on treatment) were taking calcium channel blocker. A small proportion of the participants (2.2% of hypertensive participants on treatment) were also taking ayurvedic medicine for controlling hypertension. Of those on treatment, only half had controlled hypertension (<140/90 mmHg).

### 3.3. Sociodemographic Characteristics and Hypertension

More males than females had high blood pressure (Table 2). Hypertension was also associated with age. Participants with lower educational status (primary and lower) were more likely to have hypertension. Ethnicity was also significantly associated with hypertension. However, there was no statistically significant association between occupation and hypertension.

### 3.4. Factors Associated with Hypertension

#### 3.4.1. Smoking

The proportion of current and past smokers was 19.9% and 17%, respectively. Both current smoking (*p* = 0.009) and past smoking (*p* = 0.001) participants were significantly associated with hypertension. The mean duration of smoking was also significantly higher (*p* = 0.012) in hypertensive participants than in normotensive participants (Table 3).

#### 3.4.2. Alcohol Consumption

More than one-quarter (27%) of participants reported current alcohol use. Alcohol consumption had a significant positive association (*p* = 0.035) with hypertension. The hypertensive participants reported drinking a significantly higher number of standard drinks in a single sitting (*p* < 0.001) than normotensive participants (Table 3).

#### 3.4.3. Fruit and Vegetable Consumption

Approximately, 1 in every 10 participants (11.4%) reported consuming the recommended amount of fruit and vegetable daily. The daily median intake for total fruit and vegetables per person was 188.9 gm (interquartile range (IQR): 204 gm). For green leafy vegetable, only the median intake was 75 gm per day (IQR: 125 gm). Median daily fruit intake per person was 45 gm (IQR: 120 gm). Fruit and vegetable intake was not significantly associated (*p* = 0.542) with hypertension.

#### 3.4.4. Physical Activity

More than three-quarters of participants (78.4%) had the sufficient physical activity level (≥600 METs/week). The median (IQR) of METs of moderate and vigorous physical activities per week was 1850 (IQR: 2853). Adequate physical activity was associated with normal blood pressure (*p* = 0.038).

#### 3.4.5. Obesity

The mean of body mass index (BMI) among respondents was 24.8 kg/m^2^. It was significantly different (*p* < 0.001) between people having high and normal blood pressure. Similarly, waist (*p* < 0.001) and hip (*p* < 0.004) circumferences were also significantly higher in hypertensive participants than in normotensive participants.

#### 3.4.6. Diabetes and Other Factors

One in every ten participants (10.7%) reported that they had diabetes mellitus. Similarly, 12.6% of participants had the presence of cardiovascular disease (CVD) among their first-degree relatives and 31.6% of female participants had menopausal history. Diabetes (*p* < 0.001), cessation of menstruation (*p* < 0.001), and having family history of CVD (*p* < 0.001) were significantly associated with hypertension (Table 3).

### 3.5. Adjusted Associated Factors of Hypertension

In multivariable model, BMI (*β* = 0.127 and *p* < 0.001), smoking (*β* = 0.671 and *p* = 0.005), alcohol use (*β* = 0.473 and *p* = 0.03), insufficient physical activity (*β* = 0.472 and *p* = 0.04), and presence of diabetes (*β* = 0.934 and *p* = 0.001) were identified as significant factors associated with hypertension (Table 4). Every unit gain in BMI increased the likelihood of having hypertension by 13.5%. The chance of being hypertensive among current smokers was 1.95 times higher than among nonsmokers. Similarly, current alcohol users had 60% higher chance of having hypertension than the participants who refrained from alcohol. After controlling for other factors, the odds of being hypertensive were about 60% higher for the participants who had insufficient physical activity compared to those who had sufficient physical activity. In the same way, presence of diabetes among study participants increased the odds of being hypertensive by 2.54 times compared to nondiabetic participants.

## 4. Discussion

Our study found a high prevalence of hypertension in people living in the newly declared municipalities of Kathmandu. The factors associated with hypertension in this study group were found to be smoking, BMI, alcohol use, poor physical activity, and diabetes. This was the first community-based study conducted to estimate the prevalence of hypertension and identify its associated factors in the area. However, this study could not examine causal relationship between hypertension and its risk factors because of the nature of study design.

The prevalence of hypertension in the study population (32.5%) was higher than in those of noncommunicable disease (NCD) risk factor surveys in Nepal (21.5% in 2007; 25.7% in 2013) [21, 25]. This disparity in findings may be due to differences in the study populations as the NCD surveys were conducted both in urban and in rural areas, while the current study was conducted in a periurban setting. Rural Nepal has a lower burden of hypertension than urban areas [26]. Similarly, the finding was nearly the double of the hypertension prevalence (19.7%) reported by a study conducted in similar setting of Kathmandu in 2005 [27]. This almost twofold increase in hypertension prevalence in ten years may be due to changes in living conditions and lifestyle in that time. This is further supported by the findings from a study conducted in rural areas of Kathmandu that reported a threefold increase in hypertension prevalence in 25 years [26]. This increase was attributed to increasing BMI in the study population [28].

Although it is well established that smoking increases the risk of hypertension, the strength of this association may differ between study settings and populations. Our study showed that smoking increased the likelihood of being hypertensive by twofold. This finding is consistent with previous studies conducted in India and China [29–31]. However, some observational studies reported smokers having a lower risk of hypertension than nonsmokers, which contrasted to our finding [32–37]. Our study also found that the median length of smoking duration was significantly higher among the hypertensive participants. Therefore, one of the reasons for the aforementioned inconsistency in findings could be the variations in frequency and total duration of smoking.

Our study found that the odds of having hypertension were increased by 1.6 times with the use of alcohol. The result was in line with the findings reported by Todkar et al. in India and Wei et al. in China [38, 39]. However, there are some studies that found protective effects of alcohol on hypertension [40, 41]. Some also failed to demonstrate any significant association between alcohol consumption and hypertension [30, 35]. These differences in findings might have occurred because of difference in amount and concentration of alcohol consumed. Sacco et al. and Kannel and Ellison concluded that protective effect of alcohol in hypertension and other cardiovascular diseases was mainly detected among the moderate alcohol users (up to two standard drinks per day). The effect was opposite among those consuming seven and more drinks per day [40, 41]. A meta-analysis of 15 randomized controlled trials observed a dose response relationship of alcohol reduction and on blood pressure [42]. This is supported by the current findings which demonstrated that the median number of standard drinks consumed was significantly higher among the hypertensive participants. Further research is required to explain the apparent bidirectional effects of alcohol intake on hypertension.

The current study revealed that one in every four participants reported being a current alcohol user. The proportion was higher than that of recent noncommunicable disease risk factor survey (17.4%) in Nepal [25]. This may be due to a higher social acceptance of alcohol use in the study areas compared to Nepal as a whole. The high rates of alcohol consumption in this population need to be considered when implementing programs to address hypertension.

Despite the well-recognized inverse relationship between fruit and vegetable consumption and hypertension [43, 44], our study showed no association between them. A study conducted in rural Nepal also reported a similar result [26]. A more detailed food frequency questionnaire in future studies may assist in confirming these findings. However, the fluctuating pattern of fruit and vegetable consumption in Nepal due to the seasonal availability of these foods may make clarifying the association with hypertension difficult [24]. The ideal measure of Nepalese fruit and vegetable consumption warrants further discussion.

This study found that sedentary lifestyle was an issue in this study population. The proportion of participants (21.6%) having insufficient physical activity (<600 METs/Week) was six times higher than that reported in a national survey (3.5%) [25]. Unplanned and rapid urbanization, high population density, increased use of motorized vehicles and, modern technology could be predisposing factors for low physical activity among this population [45]. The increased risk of hypertension among study participants reporting low levels of physical activity was consistent with other studies [30, 32, 45]. These findings suggest that several cardiometabolic problems may arise in near future as the consequences of insufficient physical activity. Even though confirming the causal relationship between physical activity and cardiometabolic diseases, including hypertension, is beyond the scope of this study, several previous studies have demonstrated the association [46, 47]. Therefore, Nepal should not delay initiating interventions that improve physical activity through community-based strategies that incorporate informational, behavioral, social, policy-making, and environmental approaches [48].

Body mass index, waist circumference, and hip circumference, all measures related to overweight and obesity, were significantly higher in hypertensive participants than in normotensive participants. These findings were in line with results from previous studies conducted in Nepal [26, 28]. Like physical inactivity, obesity has also well-recognized independent relationship with a spectrum of cardiometabolic disorders including hypertension [49]. There are several mechanisms hypothesized to explain the link between obesity with hypertension [10, 11, 49, 50]. It is generally thought that the accumulation of visceral and ectopic fat in a number of tissues and organs alters the metabolic and hemodynamic pathways, leading to the development of hypertension in obese people [49, 50]. The reduction of overweight and obesity by improving nutrition and increasing regular physical activity is the best way to avoid or improve hypertension [51].

Diabetes was associated with twofold increased odds of being hypertensive. The finding was consistent with the results of the studies conducted in similar settings of Kathmandu, Nepal [52, 53], and internationally [54, 55]. Hypertension is often reported to be one of the most common comorbid conditions in those suffering from diabetes. For instance, around half of diabetic patients in Australia have hypertension [56]. Similarly, almost a quarter of hypertensive patients were found to have diabetes in China and India [57, 58]. The coexistence of hypertension and diabetes might be because of sharing common risk factors like smoking, alcohol consumption, unhealthy diets, and physical inactivity. Due to nature of this study, it is difficult to state with certainty whether diabetes led to hypertension or hypertension led to diabetes. This study only included self-reported diabetic cases, which would underestimate the rate of diabetes in the study population. Further research is required in this population to accurately estimate the impact of diabetes on hypertension.

Our study also found associations between hypertension and other recognized risk factors including menopause [59–61]. Similarly, presence of cardiovascular diseases including hypertension among first-degree relatives of the participants had a significant association with hypertension among study population. The familial pattern of hypertension was well established long before [62], and it is now considered as one of the major nonmodifiable risk factors for hypertension, like age and race [63].

This study identifies some of the major factors associated with hypertension in people living in the outer areas of Kathmandu. Several of these factors could be targeted to improve the health of the population in these areas. One example where improvements have been made is in relation to smoking. The proportion of current smoking in our study (19.9%) was similar to that of a national survey (18.5%) conducted in Nepal [25]. After signing the WHO Framework Convention on Tobacco Control (WHO FCTC), Nepal is implementing tobacco control initiatives such as health warnings on tobacco products, prohibition of smoking in public places, heavy taxation of the tobacco industry, and increasing public awareness of harmful effects of tobacco. Since these initiatives have been implemented there has been a decreasing trend in prevalence of smoking in Nepal [64, 65]. The presence of large proportion of past smokers (17%) in current study might also suggest the trend of quitting smoking in the study areas. These initiatives should also lead to an improvement in the health of people in Nepal, including a reduction in the risk of hypertension. Initiatives to improve factors like physical activity and alcohol consumption in these regions of Nepal would also be useful in reducing the risk of hypertension and improving overall health.

## 5. Conclusion

Overall, this study determined a high prevalence of hypertension in the study population. Hypertension was associated with smoking, alcohol consumption, low physical activity, obesity, and diabetes. Therefore, community-based approaches for reduction of hypertension and its risk factors are essential. Effective community-based preventive and control strategies might provide the best opportunities to avoid hypertension driven health and economic consequences in Nepal.

## Figures and Tables

**Table 1:** Distribution of sociodemographic characteristics by gender.

Variables	Categories	Male	Female	Total	*p* value
		*N* (%)	*N* (%)	*N* (%)	
Age groups	<30 years	49 (20.2)	69 (20.0)	118 (20.1)	0.147
	30–39 years	47 (19.4)	90 (26.1)	137 (23.3)	
	40–49 years	69 (28.5)	81 (23.5)	150 (25.6)	
	50–59 years	37 (15.3)	63 (18.3)	100 (17.0)	
	>60 years	40 (16.5)	42 (12.2)	82 (14.0)	

Education	No formal education	29 (12.0)	143 (41.4)	172 (29.3)	<0.01
	Primary and lower	30 (12.4)	53 (15.4)	83 (14.1)	
	Secondary	64 (26.4)	65 (18.8)	129 (22.0)	
	Higher Secondary	52 (21.5)	47 (13.6)	99 (16.9)	
	Bachelor and higher	67 (27.7)	37 (10.7)	104 (17.7)	

Ethnicity	Brahman	95 (39.3)	111 (32.2)	206 (35.1)	0.246
	Chetri	68 (28.1)	103 (29.9)	171 (29.1)	
	Newar	48 (19.8)	71 (20.6)	119 (20.3)	
	Others	31 (12.8)	60 (17.4)	91 (15.5)	

Occupation	Job	66 (27.3)	35 (10.1)	101 (17.2)	<0.01
	Self-employed	87 (36.0)	54 (15.7)	141 (24.0)	
	Homemaker	30 (12.4)	212 (61.4)	242 (41.2)	
	Others	59 (24.4)	44 (12.8)	103 (17.5)	

Marital status	Unmarried	36 (14.9)	32 (9.3)	68 (11.6)	<0.01
	Married	204 (84.3)	283 (82.0)	487 (83)	
	Others (separated or widow)	2 (0.8)	30 (8.7)	32 (5.5)	

Socioeconomic status	Below poverty line	115 (47.9)	170 (49.7)	285 (49.0)	0.671
	Above poverty line	125 (52.1)	172 (50.3)	297 (51.0)	

Note: primary and lower education, grade 1–5; secondary, grade 6–10; higher secondary, grade 11-12; job, governmental or nongovernmental employment; self-employed, working for oneself as a freelancer or the owner of a business or working in own farm; homemaker, a person, especially a housewife, who manages a home; other occupations, volunteer or student or unemployed or retired; below poverty line, income less than 40,933 Nepalese Rupees per annum.

**Table 2:** Association of sociodemographic characteristics with hypertension.

Variables	Categories	Hypertension		*p* value
		Yes	No	
		*N* (%)	*N* (%)	
Gender	Male	93 (38.4)	149 (61.6)	0.011
	Female	98 (28.4)	247 (71.6)	

Age groups	<30 years	7 (5.9)	111 (94.1)	<0.001
	30–39 years	22 (16.1)	115 (83.9)	
	40–49 years	71 (47.3)	79 (52.7)	
	50–59 years	45 (45.0)	55 (55.0)	
	≥60 years	46 (56.1)	36 (43.9)	

Education	No formal education	72 (41.9)	100 (58.1)	<0.01
	Primary and lower	33 (39.8)	50 (60.2)	
	Secondary	37 (28.7)	92 (71.3)	
	Higher secondary	22 (22.2)	77 (77.8)	
	Bachelor and higher	27 (26.0)	77 (74.0)	

Ethnicity	Brahman	54 (26.2)	152 (73.8)	0.049
	Chetri	55 (32.2)	116 (67.8)	
	Newar	46 (38.7)	73 (61.3)	
	Other	36 (50.0)	3 (50.0)	

Occupation	Job	32 (31.7)	69 (68.3)	0.725
	Self-employed	43 (30.5)	98 (69.5)	
	Homemaker	85 (35.1)	157 (64.9)	
	Others	31 (30.1)	72 (69.9)	

Marital status	Unmarried	8 (11.8)	60 (88.2)	<0.001
	Married	167 (34.3)	320 (65.7)	
	Others (separated or widow)	16 (50.0)	16 (50.0)	

Socioeconomic status	Below poverty line	95 (33.3)	190 (66.7)	0.665
	Above poverty line	94 (31.6)	203 (68.4)	

Note: primary and lower education, grade 1–5; secondary, grade 6–10; higher secondary, grade 11-12; job, governmental or nongovernmental employment; self-employed, working for oneself as a freelancer or the owner of a business or working in own farm; homemaker, a person, especially a housewife, who manages a home; other occupation, volunteer or student or unemployed or retired; below poverty line, income less than 40,933 Nepalese Rupees per annum.

**Table 3:** Association of behavioral, metabolic, and other factors with hypertension.

Variables	Categories	*N*	Hypertension		Total	*p* value
			Yes	No		
			*n* (%) or mean ± SD or median (IQR)	*n* (%) or mean ± SD or median (IQR)	*n* or mean ± SD or median (IQR)	
Smoking (current)	Yes	587	50 (42.7)	67 (57.3)	117	0.009
	No		141 (30.0)	329 (70.0)	470	

Smoking (past)	Yes	470	36 (45.0)	44 (55.0)	80	0.001
	No		105 (26.9)	285 (73.1)	390	

Smoking duration among current smoker (years)	—	114	25 (24)	13.5 (20)	20 (26)	0.012^*∗*^

Alcohol consumption (current)	Yes	587	62 (39.2)	96 (60.8)	158	0.035
	No		129 (30.1)	300 (69.9)	429	

Average standard alcohol intake in single sitting	—	156	4 (6)	2 (3)	3 (4)	<0.001^*∗*^

Sufficient fruit and vegetable consumption	Yes	587	24 (35.8)	43 (64.2)	67	0.542
	No		167 (32.1)	353 (67.9)	520	

Sufficient physical activity	Yes	587	140 (30.4)	320 (69.6)	460	0.038
	No		51 (40.2)	76 (59.8)	127	

BMI (kg/m^2^)	—	587	26.2 ± 4.5	24.1 ± 4.1	24.8 ± 4.4	<0.001

Waist circumference (cm)	—	587	84 ± 11.8	78 ± 10.1	83.9 ± 11	<0.001

Hip circumference (cm)	—	587	90.5 ± 9.9	88.1 ± 8.9	90.9 ± 9.3	0.004

Diabetes mellitus	Yes	587	34 (54.0)	29 (46.0)	63	<0.001
	No		157 (30.0)	367 (70.0)	524	

Menopause	Yes	281	41 (21.4)	151 (78.6)	192	<0.001
	No		43 (48.3)	46 (51.7)	89	

CVD family history	Yes	562	37 (52.1)	34 (47.9)	71	<0.001
	No		144 (29.3)	347 (70.7)	491	

^*∗*^Mann-Whitney *U* test was used to compare nonnormally distributed data.

Note: sufficient fruit and vegetable consumption, intake of ≥400 gm of fruit and vegetable per day; sufficient physical activity, ≥600 METs of moderate and vigorous activity in a week; one standard drink, 10 grams of ethanol.

BMI, body mass index; CVD, cardiovascular disease.

**Table 4:** Multivariable analysis for hypertension.

Variables	Category	*β*	*p* value	AOR	95% CI for AOR	
					Lower	Upper
Constant		−2.220	0.005	0.109		

BMI (kg/m^2^)		0.127	<0.001	1.135	1.084	1.188

Smoking (current)	No	Reference				
	Yes	0.671	0.005	1.957	1.219	3.141

Alcohol user (current)	No	Reference				
	Yes	0.473	0.030	1.605	1.047	2.460

Physical activity	Sufficient	Reference				
	Nonsufficient	0.472	0.040	1.604	1.022	2.517

Diabetes mellitus-II	No	Reference				
	Yes	0.934	0.001	2.545	1.454	4.457

Note:  sufficient physical activity, ≥600** **METs of moderate and vigorous activity in a week.

BMI, body mass index.
